# Velocity of myosin-based actin sliding depends on attachment and detachment kinetics and reaches a maximum when myosin-binding sites on actin saturate

**DOI:** 10.1016/j.jbc.2021.101178

**Published:** 2021-09-09

**Authors:** Travis J. Stewart, Vidya Murthy, Sam P. Dugan, Josh E. Baker

**Affiliations:** 1 Department of Pharmacology, University of Nevada, Reno School of Medicine, Reno, Nevada, USA; 2 Department of Biomedical Engineering, University of Nevada, Reno, Nevada, USA

**Keywords:** myosin, actin, collective force, velocity, mechanics, TRITC, tetramethyl-rhodamine isothiocyanate

## Abstract

Molecular motors such as kinesin and myosin often work in groups to generate the directed movements and forces critical for many biological processes. Although much is known about how individual motors generate force and movement, surprisingly, little is known about the mechanisms underlying the macroscopic mechanics generated by multiple motors. For example, the observation that a saturating number, *N*, of myosin heads move an actin filament at a rate that is influenced by actin–myosin attachment and detachment kinetics is accounted for neither experimentally nor theoretically. To better understand the emergent mechanics of actin–myosin mechanochemistry, we use an *in vitro* motility assay to measure and correlate the *N*-dependence of actin sliding velocities, actin-activated ATPase activity, force generation against a mechanical load, and the calcium sensitivity of thin filament velocities. Our results show that both velocity and ATPase activity are strain dependent and that velocity becomes maximized with the saturation of myosin-binding sites on actin at a value that is 40% dependent on attachment kinetics and 60% dependent on detachment kinetics. These results support a chemical thermodynamic model for ensemble motor mechanochemistry and imply molecularly explicit mechanisms within this framework, challenging the assumption of independent force generation.

Molecular motors such as myosin and kinesin often work in groups to perform diverse biological functions such as vesicle transport, cell division, wound healing, and muscle contraction (1, 2, 3). The mechanochemistry of individual motors is in many instances well characterized (4, 5, 6, 7, 8), and determining how molecular motor mechanics scale from single molecule to ensemble mechanochemistry is the next step in understanding the macroscopic mechanics of biological systems. Our understanding of the factors that influence macroscopic mechanics is currently underdeveloped. These factors include basic relationships between motor kinetics, energetics, force generation, force transmission, compliant linkages, and external loads. The goal of this study is to better define these relationships in order to more accurately describe the emergent mechanics of molecular motor ensembles.

Optical traps and *in vitro* motility experiments have been used to study how force and motion generation change with increasing numbers, *N*, of motors (9, 10, 11) and in general show that the mechanics of many motors working together is not a simple sum of the molecular mechanics of individual motors (4, 12, 13). Consistent with the chemical thermodynamic model that we first proposed over 20 years ago (14), many studies now indicate that force is collectively generated and thermally distributed within systems of motors (12, 13, 15). This leads to emergent mechanochemical properties (12, 13, 16) that are more accurately described by the thermodynamics of a motor ensemble than by molecular mechanics (14, 17, 18).

With thousands of myosin molecules working together to generate force and movement, muscle is an ideal system in which to study emergent motor behaviors. In the 1920s and 1930s, early pioneers in biophysics like A.V. Hill and W.O. Fenn made precise measurements of muscle power and heat output (19, 20, 21) that established macroscopic energetic constraints (like muscle force) on muscle mechanics and chemistry using classical chemical thermodynamics. Since then, researchers have focused more on reductionist approaches using electron microscopy, X-ray diffraction, spectroscopic techniques, stopped flow kinetics, crystal structures, and single molecule mechanics measurements (22, 23, 24, 25, 26, 27, 28, 29) to provide detailed structural, biochemical, and mechanical descriptions of the molecules involved in muscle contraction. For example, from these studies we now know that the basic molecular mechanism for muscle contraction involves a discrete displacement of an actin filament generated by a myosin structural change induced by strong actin binding. However, despite these remarkable insights into basic molecular mechanisms, it is still unclear how these observable, simple, discrete molecular mechanisms scale up to the mechanics and chemistry of muscle in a way that is consistent with the macroscopic energetic constraints described by Hill and Fenn (19, 21) and more recently implied by our observation that the free energy for the discrete myosin working step is a function of muscle force (17).

The conventional independent force model of muscle contraction assumes that actin sliding velocities, *V*_*max*_, are limited by detachment of individual myosin motors from actin (30). However, this model does not account for the thermal equilibration of forces that exists in most chemical systems and is inconsistent with the observation that *V*_*max*_ is influenced by both actin–myosin attachment (4, 10, 16, 18, 31) and detachment kinetics (30, 32). Here we use mathematical modeling and an *in vitro* motility assay to better understand how both attachment and detachment kinetics contribute to *V*_*max*_.

In an *in vitro* motility assay, the velocity, *V*(*N*), at which actin filaments slide over a bed of myosin molecules increases with increasing numbers, *N*, of myosin molecules, saturating at a maximum velocity, *V*_*max*_, through a mechanism that continues to be disputed. For decades, it has widely been assumed that—in accord with independent force models—*V*_*max*_ is limited by what are effectively molecular mechanical barriers to force transmission between independent force generators (30, 32). Specifically, a single strongly bound myosin head is assumed to prevent the working step of other myosin heads from moving actin and transmitting forces between them, and thus movement is limited by detachment of the resistive myosin head.

To describe this hypothetical mechanical limit to *V*_*max*_, we consider the probability, *P*(*N*), that *N* myosin heads stall actin movement by myosin working steps. According to the independent force model, *P*(*N*) is simply the probability that at least one myosin head is bound to actin (32). According to a collective displacement model that we recently developed, *P*(*N*) is the probability that at least one myosin head is bound to actin and has reached the end of its mechanical tether (33). Here we develop a thermodynamic force model in which *P*(*N*) is the probability that an ensemble of myosin heads collectively reaches an internal stall force. Of importance, *P*(*N*) in the latter two models is clearly less than that in the independent force model. In all models, when *P*(*N*) = 1, actin movement can only occur with the detachment of the resistive head(s) (see Experimental procedures), at which point *V*(*N*) saturates at a *V*_*max*_ that is limited by actin–myosin detachment kinetics. Although this solid-state, detachment limit is theoretically possible within any of the above models, here we show that experimentally it is never reached (*P*(*N*) is always less than one) by myosin ensembles under physiological conditions.

We determine the chemical kinetics underlying *V*(*N*), *P*(*N*), and *V*_*max*_ using an *in vitro* motility assay to directly measure and correlate, under nearly identical conditions, the *N*-dependence of actin sliding velocities, *V*(*N*); actin-activated ATPase activity, *v*(*N*); small molecule inhibition of ATPase activity; force generation against a mechanical load, *F*(*N*); and calcium sensitivity of thin filaments, pCa_50_(*N*). In all cases, we observe that these *N*-dependent measurements saturate at an *N* similar to that at which *v*(*N*) saturates, consistent with saturation of myosin-binding sites on actin.

According to an independent force model this means that, at saturating *N*, there is an insufficient number of myosin heads for processive movement (*P*(*N*) < 1). Here we show that, according to a thermodynamic force model, a peak *V* is reached well before the detachment limit (*P*(*N*) < 1) with at least one myosin head strongly bound to actin.

Our data and analysis support a classic chemical thermodynamic framework for describing motor ensemble mechanochemistry, demonstrating that force generation is thermally equilibrated within ensemble motor systems. Here, within this formal framework, we continue to develop the first molecularly explicit models for how myosin working steps, resistive myosin heads, and external loads influence *V*(*N*) and how their relative contributions change with changes in *N*, linker compliance, and actin–myosin kinetics and energetics. These chemical thermodynamic mechanisms are broadly applicable to any molecular motor ensemble and account for our observations that both *V*(*N*) and *v*(*N*) are influenced by the strain-dependent kinetics of the myosin working step and that *V*(*N*) saturates at a *V*_*max*_ that is influenced 40% by attachment kinetics and 60% by detachment kinetics.

## Results

**Figure 1:**
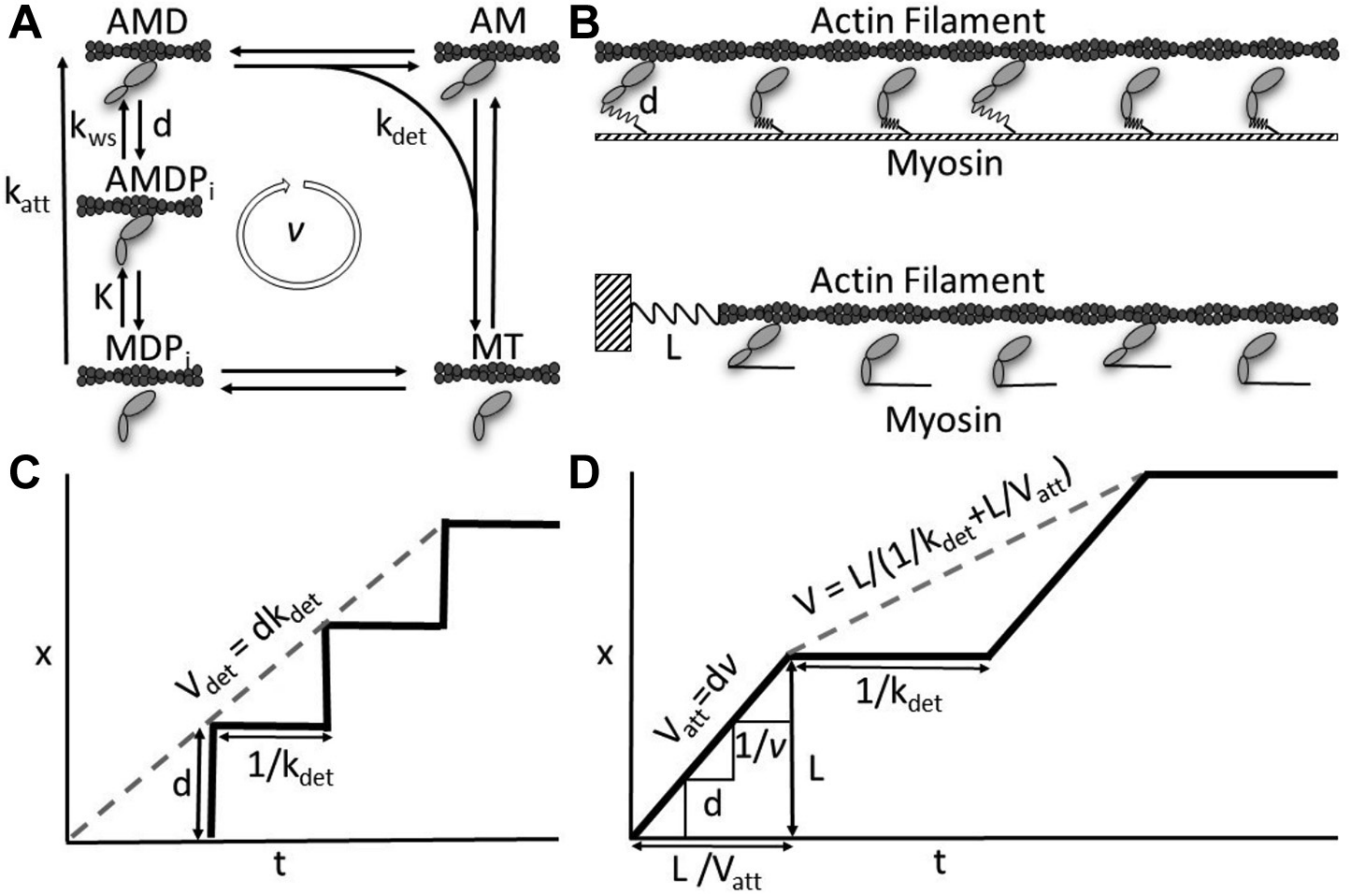
**Models for attachment- and detachment-limited myosin-based actin movement.***A*, a five-state kinetic scheme for the actin–myosin ATPase reaction. Myosin displaces an actin filament a distance *d*, with a working step (a lever arm rotation) induced by strong binding to that actin at a rate *k*_*att*_. Actin–myosin detachment occurs with ADP (D) release followed by ATP (T) binding at an overall rate, *k*_*det*_. *B*, in an independent force generator model (*top*) the working step of a myosin head generates force that is localized to that head independent of the system force. The system force is calculated as a sum of molecular forces. In a thermodynamic model (*bottom*) the working step of a myosin head generates force that equilibrates with and directly contributes to the system force. *C*, actin sliding velocities in an independent force generator model are described as the mechanical step, *d*, of a single myosin head divided by the length of time that myosin head remains bound to actin, 1/*k*_*det*_. *D*, actin sliding velocities in a thermodynamic force model are described as the distance, *L*, myosin heads (through steps of size d) collectively move an actin filament before reaching a stall force divided by the bulk (N-dependent) time it takes those myosin heads to detach from actin.

Figure 1*A* is a kinetic scheme of the actin–myosin ATPase reaction showing that the working step of a myosin head displaces an actin filament a distance *d*, upon strong actin binding at a rate *k*_*att*_, and a myosin head detaches from actin at a rate *k*_*det*_.

In an independent force model (Fig. 1*B*, top) actin sliding velocities are described in terms of the kinetics and mechanics of an individual myosin head, *V*_*max*_ = *d*·*k*_*det*_. According to this model, *V*_*max*_ is fully determined by the displacement, *d*, generated by a single myosin head and by a single rate constant, *k*_*det*_ (Fig. 1*C*), and thus *V*_*max*_ is inherently detachment limited. The *N*-dependence of *V* is determined by the probability that at least one myosin head is strongly bound to actin (*i.e.*, one strongly bound myosin head is sufficient to prevent the working step of other myosin heads from moving actin).

In a chemical thermodynamic model (Fig. 1*B*, bottom) multiple myosin heads collectively move an actin filament at *V*_*att*_ = *d*·*v* (Fig. 1*D*) where *v* is the bulk (*N*-dependent) ATPase rate. A myosin head strongly bound to an actin filament imposes a resistive but nonarresting load against actin movement, and with increasing *N* a detachment limited *V*_*det*_ = *L*·*k*_*det*_ is approached when a stall force is reached at the bulk (*N*-dependent) average maximum displacement, *L*. Movement resumes when myosin heads detach from actin at a bulk (*N*-dependent) rate (Fig. 1*D*). Figure 1*D* shows that, according to a thermodynamic model, actin sliding velocities are influenced by both attachment and detachment kinetics.

**Figure 2:**
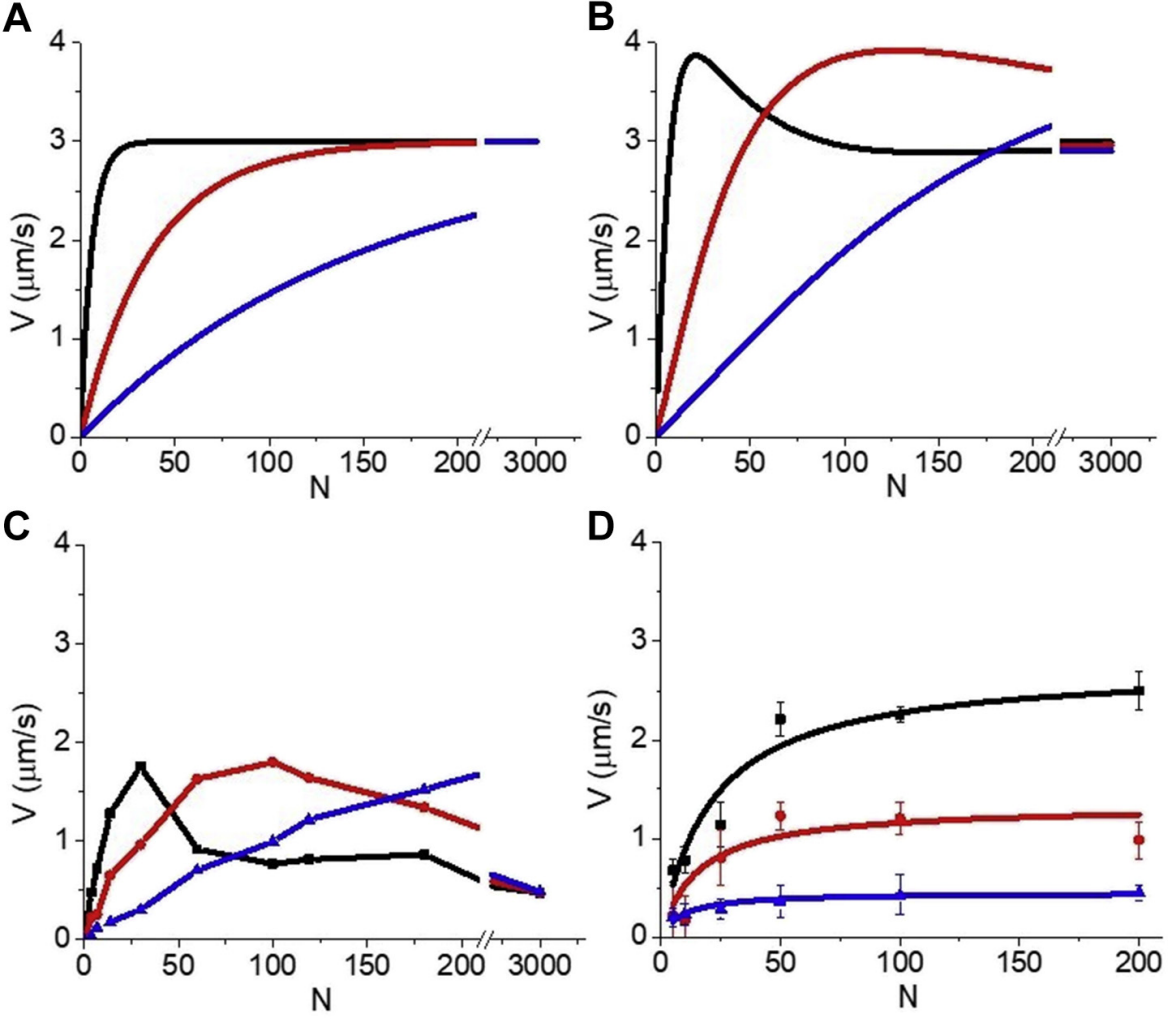
**Experimental and theoretical effects of (-)-blebbistatin on the *N*-dependence of *V*.***A*, mathematical expression for *V*(*N*) developed by Uyeda and Spudich based on the independent force model (32) with *d* = 10 nm, *k*_*det*_ = 300 s^−1^, *k*_*att*_ = 55 s^−1^ (*black lines*), 8 s^−1^ (*red lines*), and 2 s^−1^ (*blue lines*). *B*, mathematical expression for *V*(*N*) based on a collective displacement model (33) with *L* = 10 nm, *k*_*det*_ = 300 s^−1^, *d* = 10 nm, *k*_*att*_ = 55 s^−1^ (*black line*), 8 s^−1^ (*red line*), and 2 s^−1^ (*blue line*). *C*, a thermodynamic force computer simulation (see Experimental procedures) with strain-dependent, reversible kinetics and stiffness of a collective spring of 0.04 pN/nm, reverse weak-to-strong rate 0.01 s^−1^, *k*_*det*_ = 300 s^−1^, *d* = 10 nm, *k*_*att*_ = 55 s^−1^ (*black square*), 8 s^−1^ (*red circle*), and 2 s^−1^ (*blue triangle*). *D*, the effects of *k*_*att*_ on *V*(*N*) were measured in an *in vitro* motility assay using (-)-blebbistatin to inhibit *k*_*att*_. The plot shows *V* measured at different myosin surface densities (*N*) in the presence of 0 (*black squares*), 10 (*red circles*), and 50 μM (*blue triangles*) (-)-blebbistatin (decreasing *k*_*att*_) with least squares fits (*lines*) giving values for K_M_ and *V*_*max*_ of 16.1 ± 4.9 and 2.9 ± 0.3 μm/s for control, 13.3 ± 90.6 and 1.4 ± 0.3 μm/s for 10 μM, and 6.4 ± 1.3 and 0.5 ± 0.02 μm/s for 50 μM.

The *N*-dependent velocities, *V*(*N*), predicted by these two models are fundamentally different. Figure 2, *A*–*C* show the effects of attachment kinetics (*k*_*att*_ of 55, 8, and 2 s^−1^) on *V*(*N*) predicted by three models (see Experimental procedures): independent force (equation), collective displacement (equation), and thermodynamic force (discrete state simulation). According to all three models, when *N* is increased without bound (no saturation of binding sites), *V*(*N*) eventually saturates at a *V*_*det*_ that is independent of *N* and *k*_*att*_ and decreasing *k*_*att*_ increases the myosin K_M_ (*N* at half *V*_*det*_) without affecting *V*_*max*_ = *V*_*det*_.

We use an *in vitro* motility assay to directly test whether decreasing *k*_*att*_ increases K_M_ without affecting *V*_*max*_ = *V*_*det*_. Counter to predictions of all three models, Figure 2*D* shows that blebbistatin inhibition of *k*_*att*_ (34) inhibits *V*_*max*_ without increasing K_M_. This is consistent with previous studies showing that, at saturating *N*, *V*_*max*_ is influenced by *k*_*att*_ (35). These results suggest that *V*_*max*_ in a motility assay is not detachment limited (*i.e.*, is not equal to *V*_*det*_) and indicate that *V*(*N*) saturates before a detachment limit is reached (when *P*(*N*) < 1). Here we test an alternative hypothesis that *V*(*N*) saturates not at the detachment limit but with the saturation of myosin-binding sites on actin.

According to this hypothesis, *V*(*N*) and the actin–myosin ATPase rate, *v*(*N*), should exhibit similar saturation kinetics (K_M_) and correlated maximal activities (*V*_*max*_ and *v*_*max*_) (Equation 2). To test this prediction, we directly measured the *N*-dependence of both *V* and *v* in motility assays to determine *V*_*max*_ and *v*_*max*_ and the myosin K_M_ for *V* and *v* at two different ionic strengths.

**Figure 3:**
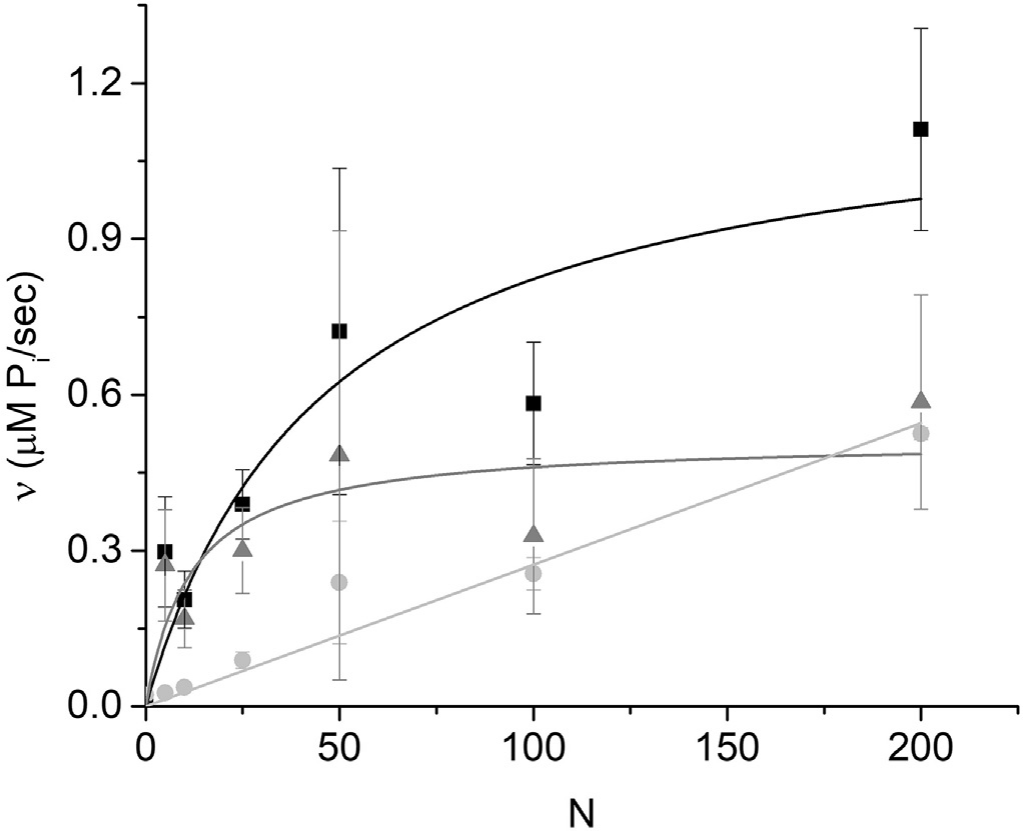
**The *N*-dependence of actin-activated ATPase activity, *v*(*N*), measured in a motility assay.** The baseline myosin ATPase activity (*light gray circles*) was measured in the absence of actin at different *N* and fit to a line (*light gray*). The ATPase activity measured in the presence of 0.15 μM actin (*black squares*) is the total ATPase activity of myosin heads interacting with actin (actin-activated ATPase) and the majority of myosin heads that are not interacting with actin (baseline myosin ATPase). Subtracting the baseline ATPase (*light gray circles*) from the total ATPase (*black squares*) gives the actin-activated ATPase rate, *v*(*N*) (*gray triangles*), which is fitted to a hyperbolic function (*gray line*).

Figure 3 shows the *N*-dependence of *v* in an *in vitro* motility assay both with and without actin filaments. Because both experiments were prepared identically with the exception of the addition of actin, the difference in these activities is the actin-activated activity. From the activities in Figure 3 and the myosin densities and flow cell geometry described (36), we estimated the baseline Mg-ATPase activity of myosin on the motility surface to be approximately 2 s^−1^, which is more than 30-fold higher than that measured in solution studies (37). This suggests that binding of myosin to the surface partially activates Mg-ATPase and/or that some of the basal activity comes from myosin in solution (not bound to the surface) that was not completely removed with the washes. Previous studies (36) have shown a linear increase in myosin ATPase activity (no actin) with increasing *N* similar to that shown in Figure 3, suggesting that saturation of the motility surface contributes to neither the saturation of *V*(*N*) nor *v*(*N*).

To maximize the *v* signal, we used higher concentrations of actin in this assay than typically used in a motility assay, and we confirmed that the majority of actin filaments were still moving under these conditions. Assuming an actin-activated ATPase activity of 40 s^−1^ (20-fold over 2 s^−1^), the ∼4-fold actin activation of ATPase activity observed at low *N* in Figure 3 suggests that ∼20% of myosin on the surface are activated by actin in this assay.

**Figure 4:**
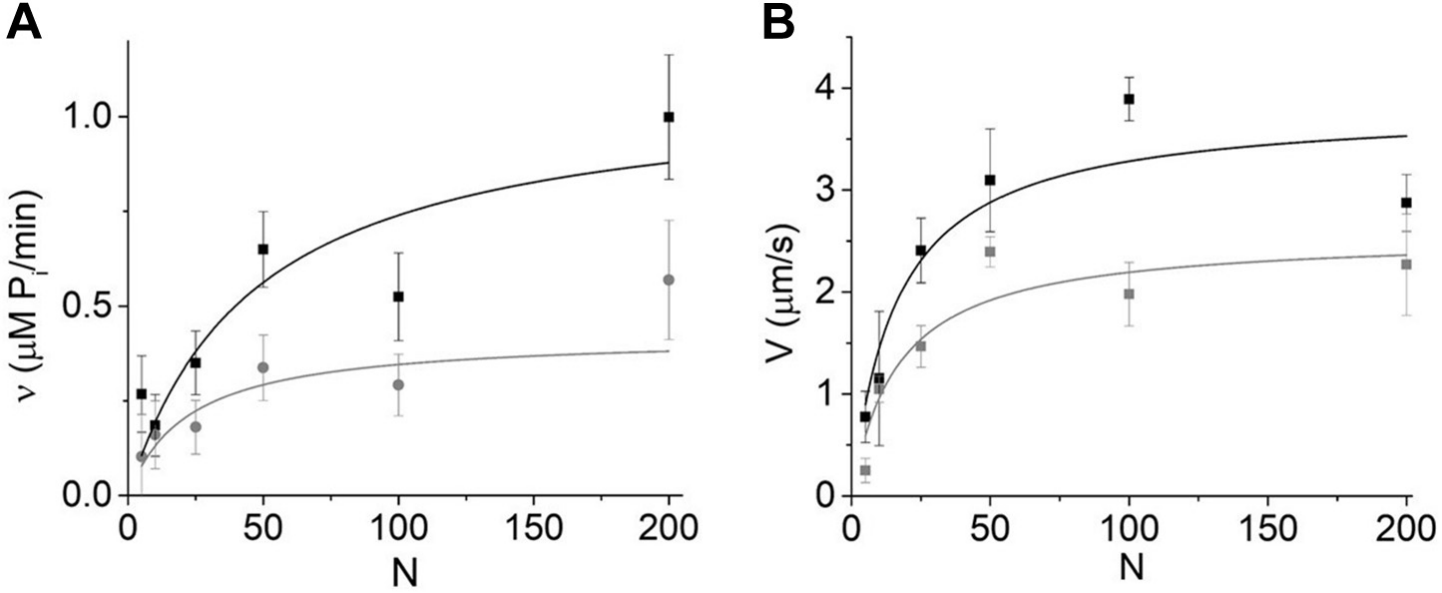
***v*(*N*) and *V*(*N*) measured at two different ionic strengths in similar *in vitro* motility assays.***A*, *v*(*N*) was measured at both 50 (*black squares*) and 100 (*gray circles*) mM KCl and fitted to hyperbolic functions (*lines*) giving K_M_ values of 46 ± 32 and 23 ± 13 for 50 and 100 mM KCl, respectively. *B*, *V*(*N*) measured under nearly identical conditions (only with 0.15 μM instead of 0.01 μM actin) to those in (*A*) at both 50 (*black squares*) and 100 (*gray circles*) mM KCl and fitted to hyperbolic functions (*lines*) giving K_M_ values of 16 ± 8 and 17 ± 9 for 50 and 100 mM KCl, respectively.

Figure 4 shows *v*(*N*) and *V*(*N*) measurements obtained in a motility assay at two different ionic strengths fit to hyperbolas. These data show that increasing KCl from 50 to 100 mM results in similar decreases in both *V*_*max*_ and *v*_*max*_ (32 ± 20% and 51 ± 28%, respectively), consistent with *V*_*att*_ influencing *V*_*max*_ (Equation 2). The observed decrease in *V*_*max*_ with increasing KCl at high ionic strength is consistent with previous studies (38). Both *V*(*N*) and *v*(*N*) exhibit similar saturation kinetics with K_M_ values of 16 ± 8 and 46 ± 32, respectively, at 50 mM KCl and 17 ± 9 and 23 ± 13, respectively, at 100 mM KCl.

To further test the saturation kinetic hypothesis and its implications for the models in Figure 2*C*, we measured the *N*-dependence of *V*(*N*) against a mechanical load. Force generation by myosin molecules along an actin filament increases linearly with the number, *N*, of myosin available to bind that actin filament. Thus, according to our hypothesis, the K_M_ for myosin force generation in a motility assay should resemble that of both *V*(*N*) and *v*(*N*) determined above. We tested this prediction by measuring the *N*-dependence of myosin force generation against a mechanical load imposed by α-actinin in a motility assay.

Alpha-actinin binds to actin and when adhered to a motility surface imposes a mechanical load against actin movement by weakly linking actin to the surface. In effect, α-actinin acts as a frictional load (39) that slows *V*. Assuming that the force, *F*(*N*), collectively generated by myosin molecules against this load increases with *N* as *F*(*N*) = *F*_*uni*_·*N*⋅*r* (where *r* is the fraction of strongly bound, force-generating myosin heads) the *N*-dependence of *V*(*N*) is described by Equation 1.

(1) $$V\left(N\right)=\left(1/\text{γ}\right)\cdot F_{uni}\cdot N\cdot r$$

where *F*_*uni*_ is the average force generated per myosin head and γ is a frictional coefficient that, according to a molecular model for friction (2), equals *N*_*α*_⋅*κ*_*α*_⋅*t*_*α*_ where *N*_*α*_, *κ*_*α*_, *t*_*α*_ are the bound number, stiffness, and bound lifetime of α-actinin molecules. According to a classical chemical thermodynamic formalism, *F*_*uni*_ = ΔG/*d*, where ΔG is the free energy for the working step (14, 17). Equation 1 is analogous to the myosin detachment-limited model illustrated in Figure 1*D*; only here at sufficiently high α-actinin concentrations *V* is influenced by α-actinin detachment kinetics. Specifically, the distance α-actinin compliant linkages are collectively displaced at stall is *L*_*α*_ = *F*_*uni*_⋅*N*⋅*r*/*N*_*α*_⋅*κ*_*α*_ and the detachment rate of α-actinin is *k*_*detα*_ = 1/*t*_*α*_. Thus, the α-actinin equivalent of the myosin detachment limited velocity illustrated in Figure 1*D* is *V* = *L*_*α*_/(1/*k*_*detα*_ + *L*_*α*_/*V*_*att*_), which at relatively high *V*_*att*_ approaches the α-actinin equivalent of Equation 2.

The collective force formalism provides a clear working-step influenced mechanism for *V*(*N*) against a mechanical load (Equation 1). This is in contrast to the independent force generator equivalent of Equation 3, which inverts the actual physical agency in this relationship. Because the independent force formalism requires that myosin heads generate force locally, myosin working steps can neither directly move actin filaments nor directly generate force, *F*(*N*), in external compliant linkages such as alpha-actinin (see Discussion); instead, the detachment-limited movement of actin subsequent to the working step stretches α-actinin linkages to generate a frictional force, *F*_*f*_. In this way a detachment-limited *V* determines *F*_*f*_ (39), and because *F*_*f*_ must be equal and opposite to the net force exerted by myosin heads (*F*_*f*_ = −*F*_*uni*_⋅*N*⋅*r*), it follows that *V* determines −*F*_*uni*_⋅*N*⋅*r*, which is simply not true. Myosin working steps actively generate *F*_*uni*_⋅*N*⋅*r* (and the opposing *F*_*f*_) against alpha-actinin linkages, and *F*_*uni*_⋅*N*⋅*r* determines *V* as described by Equation 1 (and Fig. 1*D*), not the other way around.

**Figure 5:**
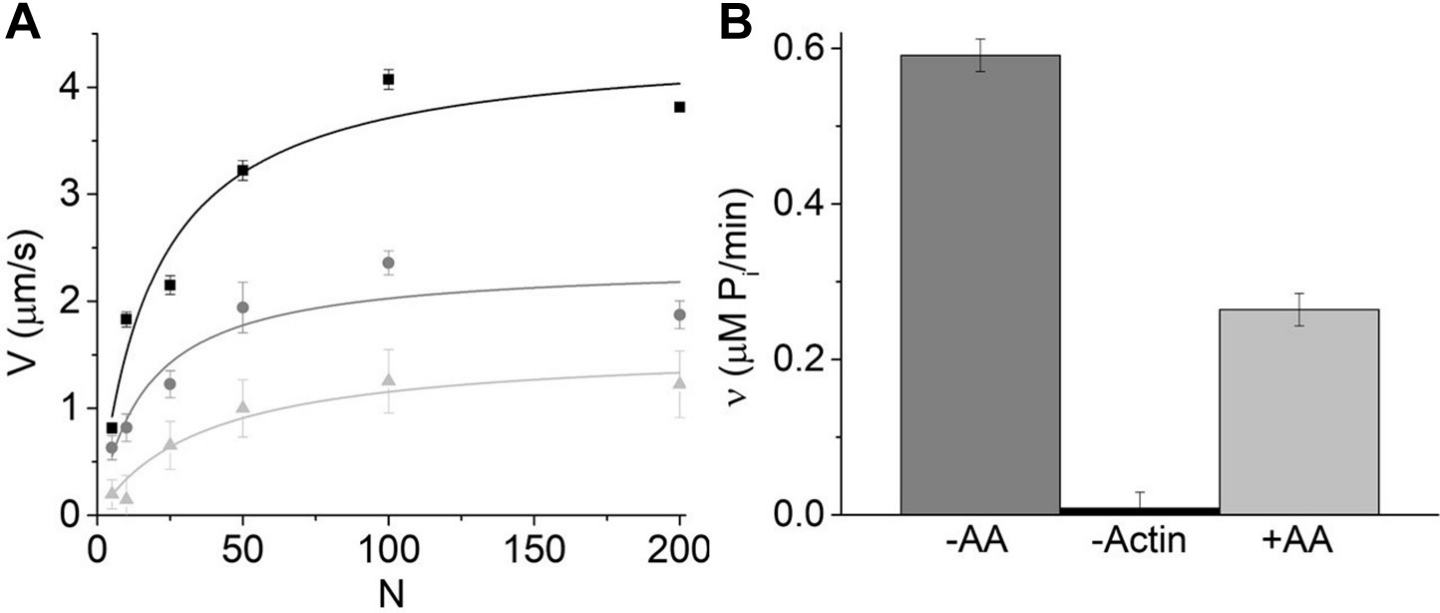
**The effects of a mechanical load on *V*(*N*) measured in an *in vitro* motility assay.***A*, *V*(*N*) measured in an *in vitro* motility assay after incubating motility flow cells with 0 (*black squares*), 0.5 (*dark gray circles*), and 1 (*light gray circles*) μg/ml α-actinin. The data were fitted to hyperbolic functions (*lines*), giving values for K_M_ of 19 ± 5, 16 ± 7, and 38 ± 13. *B*, actin-activated ATPase activity, *v*, measured in an *in vitro* motility assay (N = 5 and 1.0 μM actin) with (*light gray bar*) and without (*dark gray bar*) 1 μg/ml α-actinin shows *v* decreases from 0.59 to 0.26 μM P_i_/min upon addition of 1 μg/ml α-actinin (*p* = 0.018). The 1 μg/ml α-actinin control (myosin without actin) is indicated with the *black bar*.

Figure 5*A* is a graph of *V*(*N*) measured in an *in vitro* motility assay with and without α-actinin on the motility surface. These data show that, at subsaturating myosin (*N* < 50), *V*(*N*) slowed by an α-actinin load (increasing γ) can be recovered by increasing *N*, consistent with Equation 1. However, at *N* values above those at which *V*(*N*) and *v*(*N*) saturate, *V*(*N*) inhibited by α-actinin cannot be recovered by further increasing *N*, implying that *F*(*N*) saturates with *V*(*N*) and *v*(*N*). Fits of the α-actinin data to a hyperbolic function give K_M_ values for *F*(*N*) (16 ± 7 at 0.5 μg/ml α-actinin and 38 ± 13 at 1.0 μg/ml α-actinin) that are not significantly different from the K_M_ values for *V*(*N*) and *v*(*N*) (Table 1), further supporting our hypothesis.

**Table 1:** Summary of parameters determined in figures

Experiment	K_M_ for V	K_M_ for v
50 mM KCl	16 ± 8	46 ± 32
100 mM KCl	17 ± 9	23 ± 13
0.5 μg/ml α-actinin	16 ± 7	
1.0 μg/ml α-actinin	38 ± 13	
Actin breaking rate	13 ± 11	
pCa_50_ 50 mM KCl	24	
pCa_50_ 100 mM KCl	26	

Because the independent force model requires that myosin working steps generate force locally, strain-dependent kinetics of the working step, *k*_*ws*_, can in theory only be a function of local strain, independent of the external alpha-actinin load. In contrast, we previously showed that working step energetics are a function of an external muscle load (17), and the collective force model we developed to account for that observation predicts that *k*_*ws*_ = *k*_*ws*_°⋅exp(−*w*/k_B_T), where *w* is the work performed in collectively stretching external compliant linkages like those introduced by alpha-actin (14).

According to Equation 2, the slope of the low *N* data in Figure 5*A* is *V*/*N* = *k*_*att*_ˑ*d*. The addition of 1.0 μg/ml α-actinin decreases this slope by 73% (Fig. 5*A*), suggesting that α-actinin decreases *k*_*att*_ by decreasing the rate-limiting *k*_*ws*_. This interpretation is supported by the data in Figure 5*B*. We measure the actin-activated ATPase activity with and without α-actinin during a motility assay at low *N* (= 5), conditions under which *V* is primarily limited by *k*_*att*_. We observe that the load imposed by 1.0 μg/ml α-actinin inhibited actin-activated ATPase activity by 55% (Fig. 5*B*), consistent with the external α-actinin load inhibiting *k*_*ws*_, as predicted by collective force models.

**Figure 6:**
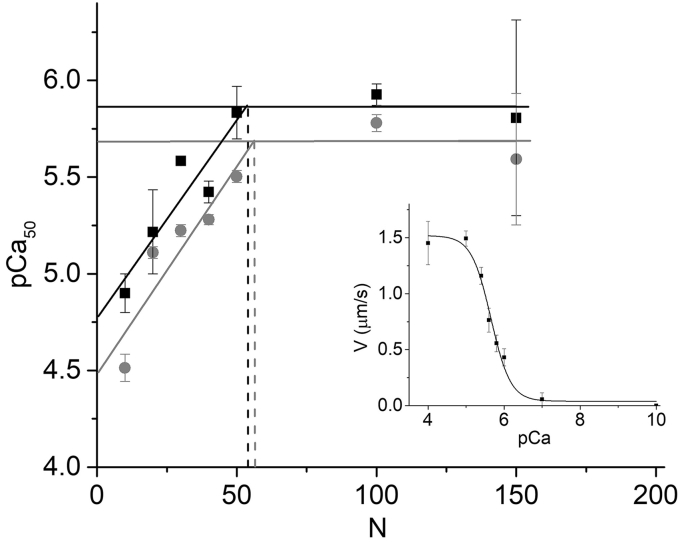
***N*-Dependence of pCa**_**50**_**in a motility assay.** The calcium dependence of thin filament sliding velocities was measured in an *in vitro* motility assay, and the data were fit to a Hill equation to obtain the calcium concentration at half-maximal activation reported as the pCa_50_ (*inset*) as previously described (41). These experiments were repeated at different *N* to obtain pCa_50_(*N*) at both 50 (*black squares*) and 100 (*gray circles*) mM KCl. The data at or below N = 50 were fit to lines with y-intercepts of 4.8 and 4.5 and slopes of 0.02 and 0.02 for 50 and 100 mM KCl, respectively. The data above *N* = 50 were averaged (*horizontal lines*) to estimate maximum pCa_50_ values of 5.9 and 5.7 at 50 and 100 mM KCl. The *N* at saturation is the intercept of the maximum pCa_50_ and the linear fit, and the pseudo K_M_ is half the *N* at saturation.

To further test the kinetic saturation hypothesis, we consider the *N*-dependence of myosin activation of thin filaments. In 2010 we developed and tested experimentally a simple two-state model for thin filament activation of thin filament motility by calcium and myosin (9, 40, 41). Our simulations and experimental data imply a simple relationship between pCa_50_ (the calcium concentration at half-maximal activation), *N*, and the actin–myosin duty ratio, *r* (the fraction of time myosin spends strongly bound to an actin filament). Specifically, we showed that pCa_50_ is proportional to *N*ˑ*r*. Previously we showed that pCa_50_ increases linearly with *N* (40, 41) up to *N* = 100 (100 μg/ml myosin incubation), but we never measured pCa_50_ at *N* > 100. Here, using an *in vitro* motility assay, we measured the calcium dependence of *V* at both 50 and 100 mM KCl and obtained pCa_50_ values from Hill fits to pCa–*V* curves (Fig. 6, inset) as previously described (41). We repeated these experiments at different *N* up to 150. Figure 6 shows that pCa_50_ values saturate at high myosin densities, which according to our model indicates that the number of myosin, *N*, available to strongly bind and activate a thin filament saturates at *N* values similar to those that saturate *V*(*N*), *v*(*N*), and *F*(*N*).

A hyperbolic fit is not well constrained by these data because the y-intercept is non-zero, and so we fit data obtained at N < 100 to a line (our two-state model) where according to our model the y-intercept is the pCa_50_ for calcium binding to TnC in the absence of myosin and the slope is proportional to the actin–myosin duty ratio (40). The horizontal lines in Figure 6 are the average pCa_50_ values measured at or above *N* = 100. The *N* at saturation was determined from the intercept of the linear fit and the average saturated pCa_50_ value, and the *N* at half saturation (a pseudo K_M_) is half the *N* at saturation. Using this approach, the pseudo K_M_ for pCa_50_ is 24 for 50 mM KCl and 26 for 100 mM KCl, similar to K_M_ values for *V*(*N*), *v*(*N*), and *F*(*N*) (Table 1).

**Figure 7:**
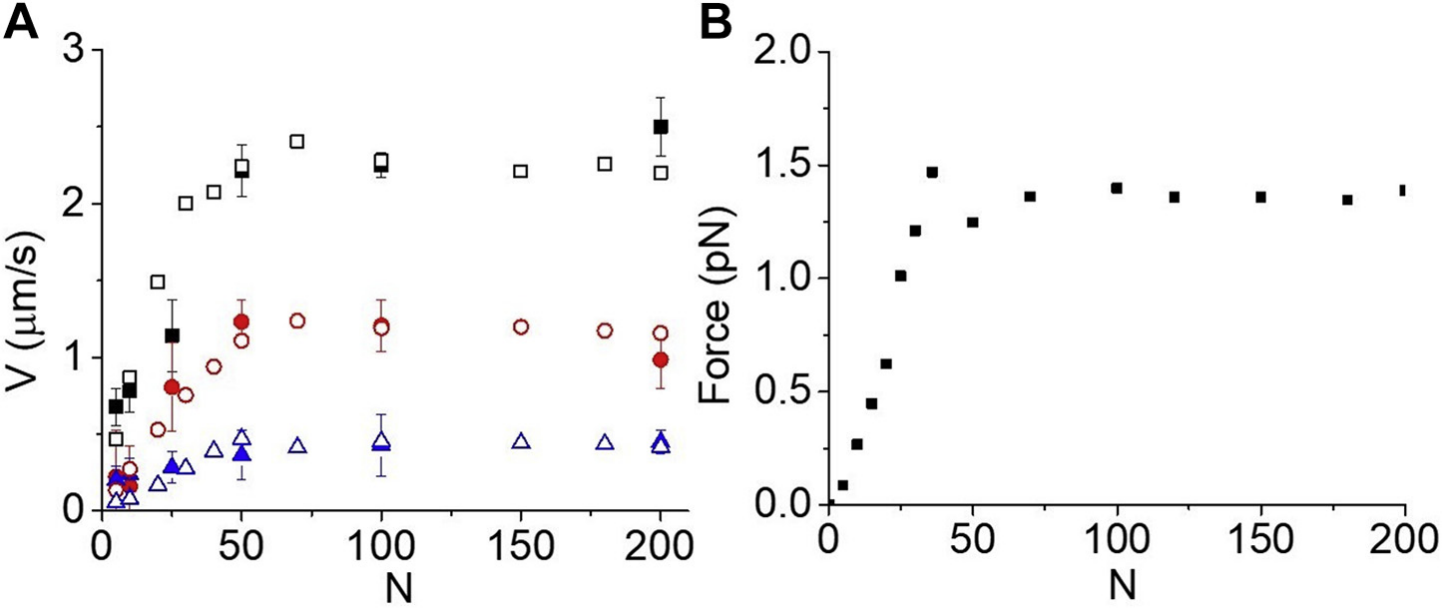
**Collective force model with saturation kinetics.***A*, computer simulations of *V*(*N*) obtained at different *k*_*att*_ values based on a collective force model with saturation attachment kinetics (*open symbols*) are overlaid with *V*(*N*) data from Figure 2*D* obtained at different blebbistatin concentrations (*solid symbols*). Parameters of the simulation are *d* = 10 nm, *k*_*det*_ = 400 s^−1^, and *k*_*att*_ of 30 s^−1^ (*black symbols*), 5 s^−1^ (*red symbols*), and 1.5 s^−1^ (*blue symbols*). *B*, in computer simulations of *F*(*N*) based on a collective force model with saturation attachment kinetics, force is generated collectively by myosin heads when they displace a single mechanical spring with spring constant κ = 0.04 pN/nm. *F*(*N*) increases linearly with *N* and saturates at the same *N* as *V*(*N*) when myosin heads saturate.

Figure 7 shows collective force computer simulations with a finite number of myosin-binding sites per micron actin and an infinite *K*. In Figure 7*A*, simulations of *V*(*N*) at different *k*_*att*_ values are compared with the blebbistatin data from Figure 2*D*. Figure 7*B* shows simulations of *F*(*N*). Although these results suggest that in a standard motility assay actin–myosin binding saturates at a *V*_*max*_ prior to *P*(*N*) saturating at 1, *P*(*N*) is not zero at *V*_*max*_. In other words, detachment kinetics still contributes to *V*_*max*_. This is supported by numerous studies that have demonstrated a correlation between *V*_*max*_ and *k*_*de*t_ (42). The extent to which *V*_*max*_ is influenced by *V*_*det*_ depends on experimental conditions, which is to say many factors influence *P*(*N*). For example, we have previously shown that *V*_*max*_ is influenced more by *V*_*det*_ in a myosin monomer–based motility assay than in a myosin filament–based motility assay (10, 33), and we have shown that *V*_*max*_ is influenced more by *V*_*det*_ at low [ATP] than at high [ATP] (16).

**Figure 8:**
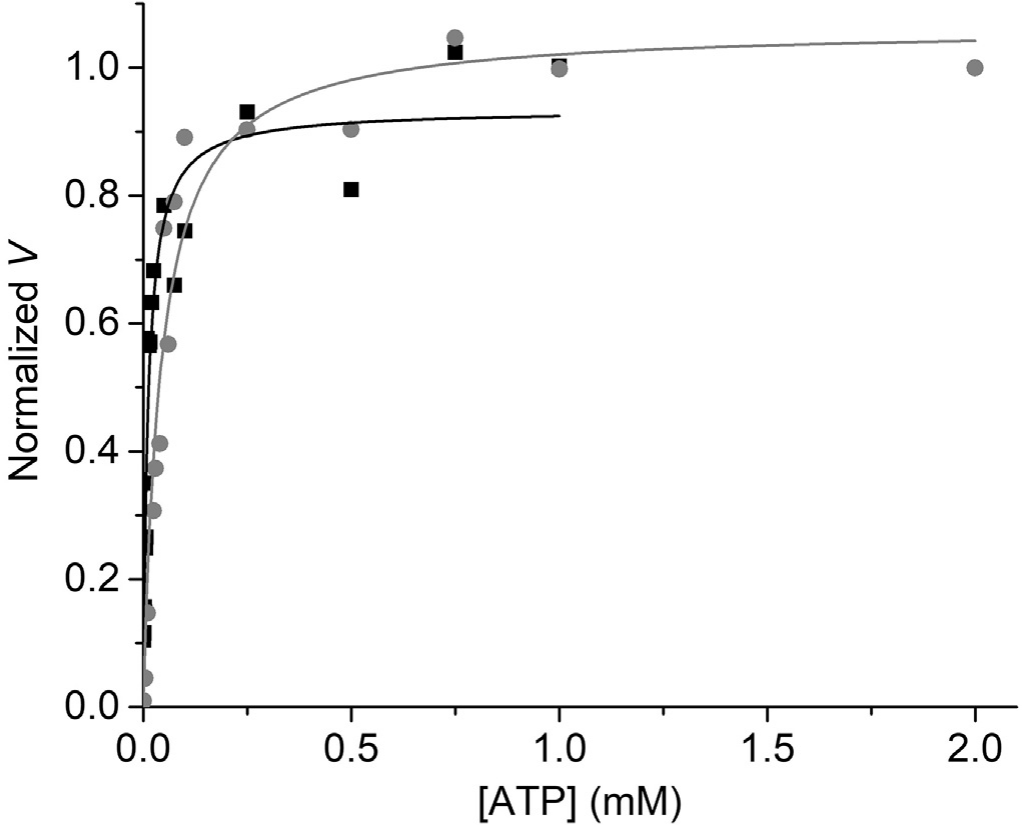
**ATP dependence of *V* at 5 and 100 μg/ml myosin.** The [ATP] dependence of V at high (100 μg/ml; *gray circles*) and low (5 μg/ml; *black squares*) myosin fitted to a hyperbola (*lines*), giving *K*_*M*_ values of 0.01 ± 0.002 at 5 μg/ml and 0.04 ± 0.007 at 100 μg/ml.

Here we estimate the extent to which *V*_*max*_ is influenced by *V*_*det*_—the *P*(*N*) when actin–myosin binding saturates—in a standard motility assay. At *P*(*N*) = 0, *V* = *V*_*att*_ is attachment limited (Equation 2) and the [ATP]-dependence of *V* has a K_M(ATP)_ ([ATP] at half *V*_*max*_) of *k*_*att*_/*k*_*T*_, where *k*_*T*_ is the second-order ATP binding constant. Figure 8 (gray circles) shows the [ATP] dependence of *V* measured in an *in vitro* motility assay at low *N* (= 5) and low *P*(*N*). A hyperbolic fit to these data gives a K_M(ATP)_ = *k*_*att*_/*k*_*T*_ of 0.01 mM. At *P*(*N*) = 1, *V* = *V*_*det*_ is detachment limited (Equation 3) and the [ATP]-dependence of *V* has a K_M(ATP)_ of *k*_*−D*_/*k*_*T*_, which is (*k*_*−D*_/*k*_*att*_)-fold greater than the K_M(ATP)_ at *P*(*N*) = 0. Here *k*_*−D*_ is the rate constant for ADP release. Assuming a duty ratio of *k*_*att*_/*k*_*−D*_ = 0.1, we expect the K_M(ATP)_ for *V*_*det*_ to be 10-fold greater than the K_M(ATP)_ for *V*_*att*_. Figure 8 (black squares) shows the [ATP] dependence of *V* obtained at saturating *N* (= 100) fitted to a hyperbola with a K_M(ATP)_ of 0.04 mM. This is 40% of the K_M(ATP)_ predicted for *V*_*det*_, implying that *P*(*N*) = 0.4 at saturating *N*.

## Discussion

Many biological processes rely on collections of molecular motors to generate macroscopic forces and movement (1, 2, 3). The mechanics and chemistry of different molecular motors have been well characterized at the level of a single molecule (4, 5, 6, 7, 8) often with an expectation that single motor mechanics will directly translate to ensemble motor mechanics to reveal the molecular mechanisms of macroscopic biological processes. This expectation is largely fueled by the independent force generator model (30) that describes ensemble motor mechanics as the sum of its molecular mechanical parts. However, data presented here and elsewhere imply that forces are collectively generated and thermally equilibrated within motor systems, leading to emergent behaviors that are not readily inferred from single molecule measurements (14, 17, 18). A system in which forces are thermally equilibrated is best described by Gibbs’ chemical thermodynamics (43), and here we continue to develop the first explicit models for *V*(*N*) and *F*(*N*) within this classical framework.

In an *in vitro* motility assay, myosin-generated actin sliding velocities, *V*(*N*), increase with increasing *N*, saturating at a *V*_*max*_ that is influenced by both actin–myosin attachment and detachment kinetics. The conventional assumption of a detachment-limited *V*_*max*_ is based more on adherence to the formal constraints of the independent force model than on experimental data, as the influence of *v* on *V*_*max*_ was long ago demonstrated (31).

Not only is the independent force model not incontrovertible but it also challenges Gibbs’ classical chemical thermodynamics. T.L. Hill developed the independent force formalism in the 1970s (44) specifically to address the problem that the assumption of independent force generation (30) is inconsistent with classical chemical thermodynamics. For the past 20 years we have argued that instead of abandoning chemical thermodynamics a different assumption was needed (14); specifically, that force generated by a myosin head is equilibrated with the force of the muscle system. Although evident and principled, this argument has been challenged (45, 46) and otherwise simply disregarded. Here, we highlight the fundamental differences between these two formalisms.

The independent force generator model (30) assumes that force generated with a myosin working step is locally equilibrated (47), which means that multiple myosin heads through their working steps cannot collectively generate force in external compliant elements (they generate force independently). Placed in a broader context, this model assumes that myosin force generation within the macromolecular assembly of multiple actin-bound myosin heads does not equilibrate within the assembly but instead equilibrates within a single protein (myosin) component of that assembly. This highly unconventional view requires an unconventional chemical thermodynamic formalism. Because Gibbs free energies describe the energetics of a system within which molecular forces are equilibrated, T.L. Hill developed a new form of chemical thermodynamics to describe molecular free energies of individual proteins thermally isolated from the assembly with which they interact. The independent force definition of *P*(*N*)—that a single actin-bound myosin head prevents actin movement and force transmission—is the purported mechanism by which myosin heads are thermally isolated in shortening muscle despite there being no known molecular mechanisms for such a barrier to thermal force. Within this framework, motor kinetics and energetics are only influenced by the work individual motors perform locally and are uncoupled from any work performed on external compliant elements through subsequent actin movement. Thus, unique to this model is the definition of molecular stress-strain curves, a characteristic feature that identifies most models of muscle contraction to date as independent force generator models (48, 49, 50, 51).

In 1999, we directly measured mechanochemical coupling in isometric muscle and observed that the free energy for the myosin working step varies proportionally with muscle force, implying that the myosin working step equilibrates with muscle force (17). This led us to develop a thermodynamic model of muscle force (14) that predicts that a myosin working step (generated by a discrete myosin lever arm rotation induced by strong actin binding) directly moves a given actin filament and directly displaces and generates force in all crossbridges, compliant linkages, and external loads that oppose that movement (16). Here, the Gibbs free energy and rate constant for the myosin working step, *k*_*ws*_, are functions of the average work, *w*, performed on all external loads and compliant linkages stretched by that transition, *e.g.*, *k*_*ws*_ = *k*_*ws*_°·exp(−*w*/k_B_T) (14, 43), consistent with the observed effects of an external α-actinin load on *k*_*ws*_ in Figure 5.

The Gibbs’ chemical thermodynamic and Hill’s molecular mechanics formalisms are fundamentally different. They are based on fundamentally different assumptions with fundamentally different formal constraints, resulting in fundamentally different predictions. The data and analysis herein highlight some of those differences and further demonstrate that motor ensemble mechanochemistry is consistent with Gibbs’ chemical thermodynamics not Hill’s molecular mechanics.

The observation that *k*_*ws*_ contributes to *V*_*max*_ implies that myosin working steps directly contribute to actin movement and perform work on all external compliant elements stretched by that movement, challenging the independent force generator formalism. The observation that myosin-binding sites on actin saturate before a detachment limit is reached implies that *k*_*ws*_ contributes to *V*_*max*_ in any actin–myosin system, including muscle. The data and analyses herein infer explicit mechanisms for a classical chemical thermodynamic model. Equations 2 and 3 along with collective force definitions of *P*(*N*) and *L* (see Experimental procedures) provide a clear description of how myosin working steps and resistive myosin heads both contribute to *V*_*max*_ and how these contributions change with *N*, actin–myosin attachment and detachment kinetics, and linkage compliance as opposed to the relatively rigid independent force prediction that *V*_*max*_ depends only on two parameters. The working step contribution to movement and force generation also provides a clear mechanism for *V*(*N*) against a load (Equation 1) as opposed to the convoluted inverse agency predicted by independent force models (see Results).

The observation in Figure 5 that an alpha-actinin load in a motility assay similarly slows *V*(*N*) (limited by *k*_*att*_ at low *N*) and *v*(*N*) (limited by *k*_*att*_) implies that the work performed by myosin working steps in stretching α-actinin linkages slows *k*_*ws*_. Although we cannot rule out that the α-actinin load diminishes to some extent *d* and/or *k*_*det*_, the observation that it decreases *k*_*ws*_ by more than 50% (Fig. 5*B*) is inconsistent with the independent force model, whereas this external strain dependence is predicted by a collective force model (18).

In the same way that the independent force model formally precludes myosin working steps from collectively performing work against an external load (17) and stretching external alpha-actinin linkages (Fig. 5, Equation 1), it formally precludes myosin working steps from collectively stretching compliant linkages associated with other strongly bound myosin heads. In contrast, in a thermodynamic force model, myosin working steps can collectively generate force by stretching compliant linkages associated with other strongly bound myosin heads, analogous to how force is collectively generated in muscle (17) or in stretching alpha-actinin linkages (Fig. 1*D*, Equation 1). Thus, one myosin head strongly bound to actin is not sufficient to stall actin movement. Instead, actin movement stalls when compliant linkages associated with strongly bound myosin heads are collectively displaced until a stall force is reached at a net displacement, *L*. *P*(*N*) is then the probability that a stall force is reached by *N* myosin heads, which occurs when many myosin heads are strongly bound to actin (Fig. 2*C*).

We have previously shown that many factors influence *L* and *P*(*N*), including the Gibbs free energy for the working step (16), the number of strongly bound heads resisting movement (16), and the compliance of resistive linkages (33). Our recent observation that the myosin S2 tether dramatically increases *L* (33, 52) allowed us to develop an idealized collective displacement model (linkages with infinitely high compliance and a hard stop at a displacement *L*) from which we developed an analytical expression for *P*(*N*). In general, however, a more realistic model requires strain-dependent kinetics and collective force generation in stretching compliant linkages. Here we have developed the simplest possible discrete state collective force model (see Experimental procedures) in which all resistive linkages are treated as a single effective spring with an effective spring constant, κ (Fig. 2*C*). It is worth noting that our computer simulations of collective force in Figure 2*C* resemble our mathematical model of collective displacements plotted in Figure 2*B*. We continue to refine and test this model through experiments like those presented here.

Our observation that *P*(*N*) = 0.4 when myosin-binding sites on actin are saturated (Fig. 8) is also inconsistent with the independent force model for *P*(*N*). Assuming that there are 28 myosin-binding sites on actin along the pseudo repeat of a 1-μm actin filament and a duty ratio of 0.1, we would expect on average 2.8 strongly bound myosin heads not 0.4 as inferred by the independent force model. In muscle, this discrepancy is even greater considering the myosin S2 tether further decreases *P*(*N*) (33). In addition, because the independent force definition of *P*(*N*) is the probability that at least one myosin head is strongly bound to actin, the fraction of time no myosin heads are strongly bound to actin is [1 − *P*(*N*)]. This would imply that a 1-μm actin filament sliding at *V*_*max*_ in a motility assay spends 60% of its time with no myosin heads strongly bound and even more time with no heads strongly bound in muscle, which is inconsistent with sustained muscle contractions. Clearly, the probability that actin-bound myosin heads stall actin movement, *P*(*N*), is much smaller than the probability that at least one myosin head is strongly bound to actin, which is to say that a strongly bound myosin head does not prevent actin movement or force transmission; myosin working steps directly move actin filaments and generate force in external elements; and multiple myosin heads collectively generate force against common actin-bound myosin heads, compliant linkages, and external loads that resist that movement, inconsistent with independent force generation.

The extent to which detachment kinetics influences *V*_*max*_ has mechanistic significance. For example, near the attachment limit [*P*(*N*) << 1], muscle shortens at a maximum velocity per myosin head with minimal energy lost to myosin head–head interactions and internal force generation, whereas near the detachment limit [*P*(*N*) = 1] myosin head–head interactions generate internal strain during shortening that can provide functionally significant cooperative mechanisms involving strain-dependent kinetics (8, 30, 53).

Similarity between the K_M_ for myosin reported here and the number of myosin-binding sites along the pseudo repeat of a 1-μm actin filament (see Results) implies that the weak-binding affinity of myosin for actin is high. Increasing ionic strength should decrease this weak-binding affinity, resulting in an increase in the K_M_ for myosin; however, a significant increase in K_M_ with addition of 50 mM KCl was not observed in Figure 4. This could be because the change in K_M_ is relatively small and within the error of our measurements. It could also be because ionic strength affects other kinetic steps in the reaction cycle that result in an offsetting decrease in the apparent K_M_.

The data and analysis reported herein provide explicit mechanisms for our observations that *V*(*N*) and *v*(*N*) are both strain dependent and that *V*(*N*) saturates at a *V*_*max*_ that is influenced 40% by attachment kinetics and 60% by detachment kinetics, filling an important gap in our basic understanding of ensemble actin–myosin mechanics. The observation that myosin-binding sites on actin saturate prior to a detachment limit precludes a detachment-limited *V*_*max*_ in any actin–myosin system, including muscle. The impact of these results extends well beyond this basic observation, however. These results inform our continuing development of the first explicit models for *V*(*N*) and *F*(*N*) within a classical chemical thermodynamic framework that is broadly applicable to any molecular motor ensemble, providing insights into how motor working steps, resistive motors, and other resistive external loads all influence and contribute to *V*(*N*) and how the relative contributions of these mechanisms change with changes in *N*, motor kinetics and energetics, and linker compliance.

## Experimental procedures

### Protein preparations

Skeletal muscle myosin was prepared from rabbit psoas muscle as described and stored in glycerol at −20 °C (54, 55). F-actin was purified from rabbit psoas muscle and stored on ice at 4 °C (56). To stabilize and label actin for *in vitro* motility assays, actin (1 μM in actin buffer) was incubated with 1 μM tetramethyl-rhodamine isothiocyanate (TRITC)-phalloidin (Sigma) overnight at 4 °C prior to dilution to the experimental concentration (see below). Skeletal tropomyosin and troponin (Tm-Tn) were purified as described (57, 58). Regulated thin filaments were reconstituted by combining 250 nM Tm and Tn to 0.015 μM TRITC-actin and incubating on ice for 20 min as described (41).

### Buffers

Myosin buffer contained 300 mM KCl, 25 mM imidazole (pH 7.4), 1 mM EGTA, 4 mM MgCl_2_, and 10 mM DTT. Actin buffer contained 50 mM KCl, 50 mM imidazole (pH 7.4), 2 mM EGTA, 8 mM MgCl_2_, and 10 mM DTT. Motility buffer contained 50 mM imidazole (pH 7.4), 2 mM EGTA, 8 mM MgCl_2_, 10 mM DTT, 0.5% methylcellulose and KCl, and 1 mM ATP. Motility buffer also contained an oxygen scavenger system (stock [c] of 2.9 mg/ml glucose, 1.6 mg/ml glucose oxidase, 2.3 mg/ml catalase) that was added immediately prior to imaging (59).

### *In vitro* motility assays

Velocities of TRITC-labeled actin filaments were measured at 30 °C as they moved over surface-attached monomeric skeletal muscle myosin. Flow cells were made by attaching a nitrocellulose-coated coverslip to a microscope slide with double-sided ¼-inch-thick tape (3M). Myosin solutions of different concentrations in myosin buffer were applied to the flow cell followed by 2 × 50-μl aliquots in sequence of 5 mg ml^−1^ bovine serum albumin in actin buffer, 10 nM TRITC-actin in actin buffer prepared as described above, actin buffer, and motility buffer. Each solution was incubated in the flow cell for 1 min before adding the next. Blebbistatin experiments were performed using 10 and 50 μM (-)-blebbistatin (Sigma-Aldrich) in the motility buffer (60). For loaded motility assays, α-actinin (Sigma Aldrich) in actin buffer was added to the flow cell following the addition of myosin at the indicated concentrations in 2 × 50-μl aliquots with 1-min incubation. Calcium experiments were performed by adding calcium to the motility buffer at concentrations determined from an algorithm based on Fabiato and Fabiato to obtain the free calcium concentrations reported here as pCa (61). A total of three experiments (N = 3) were averaged per data point. Motility assays were performed using a Nikon TE2000 epifluorescence microscope, and images were digitally acquired with an Andor iXon Ultra camera (Oxford Instruments).

### Tracking and image analysis

MetaMorph software (Molecular Devices) was used for image acquisition. For each flow cell, 30-s image sequences from three different fields were recorded. Actin velocities were manually tracked using the MTrackJ plug-in for ImageJ (NIH). Only smoothly moving filaments with trajectories greater than 3 μm were selected for analysis. An average velocity for a given trajectory meeting the above criteria was determined using ImageJ. For a given experiment (one flow cell) 45 to 100 trajectories were recorded and analyzed (n = 1). Reported *V* values are averages of at least three (n = 3) independent experiments with standard error reported with error bars.

### Measurement of actin-activated ATPase activity, *v*, in an *in vitro* motility assay

Actin-activated ATPase activity, *v*, was measured in a motility flow cell having the geometry described above with three minor differences: (i) tape was added to only one side of the flow cell to allow access to the solution within, (ii) higher concentrations of TRITC-actin (see Figure legends) were used to increase the ATPase signal, and (iii) ATPase measurements were made over longer periods than motility measurements. Specifically, solutions and proteins were added as described above for the motility assay, but instead of mounting the slide on a microscope, slides were placed on a slide warmer set to 30 °C. At 5, 10, 15, 30, 35, 45, and 60 min time points, an individual flow cell was opened (side without the tape was lifted) and 25 μl of solution was removed and added to 250 μl of malachite green (20 ml 0.045% malachite green, 6.6 ml 1% sterox, and 2.2 ml 4.2% ammonium molybdate) and mixed with a positive displacement pipette. The reaction was quenched with 30 μl of 34% sodium citrate solution, and absorbance was read at 650 nm in a 96-well plate reader using Gen5 (Biotek) software. In all experiments, the time course of absorbance was well fit to a line with the slope being a proportional measure of the total ATPase activity in that flow cell. Because the total ATPase activity is the sum of the ATPase activity of myosin heads interacting with actin (actin-activated ATPase) and the activity of myosin heads not interacting with actin (basal ATPase), we subtract the basal ATPase from the total ATPase to obtain the actin-activated ATPase activity. We measured the basal (myosin) ATPase activity for each experimental condition by repeating all of the steps above except no TRITC-actin was added. Because we were unable to quantitate the amount of actin interacting with myosin in these experiments, we were only able to measure relative changes in actin-activated ATPase activity not absolute values. One-hour time courses obtained both in the presence and absence of actin constitute one experiment (n = 1), and a total of three experiments (N = 3) were averaged per data point.

### Calculating *N*

Based on previous studies (36) and basal myosin ATPase data in Figure 3, we assumed a linear relationship between the surface density of myosin on a motility coverslip and the myosin concentration, [M], used to incubate a flow cell for 2 min. The number, *N*, of myosin available to bind a unit length (1 μm) of actin is proportional to the myosin surface density. To calculate *N*, we multiply [M] by a conversion factor 1 (myosin per μm actin) (ml/μg) consistent with estimates in Harris and Warshaw (36). Within multiple experiments performed within days of each other, variability in *N* can occur with a combination of very subtle changes in temperature, denaturing of protein over days, differences in flow cell preparation, etc. Because we performed our experiments within days of each other, we estimated variability in *N* by measuring the intensity of fluorescently labeled myosin on a coverslip among different coverslips at different *N* values on the same day. We measured a standard deviation in fluorescence intensity for all *N* of less than ±80%. This error would contribute to the measured error in *V* over the range of *N* where *V* increases with *N* and would be reflected in the reported error in K_M_ (Table 1).

### Molecular models for *V*(*N*)

Actin and myosin catalyze the hydrolysis of ATP through intermediate steps illustrated in Figure 1*A*. Upon myosin weak-to-strong binding to actin and release of inorganic phosphate, P_i_ (24), a discrete rotation of the myosin lever arm displaces an actin filament a distance *d* (28, 62, 63). This mechanochemical step, which we refer to as the working step, occurs at a rate *k*_*ws*_. The effective attachment rate, *k*_*att*_, includes the weak association of actin and myosin, *K*, that precedes *k*_*ws*_, or *k*_*att*_ = *K*·*k*_*ws*_. Following ADP release and ATP binding, myosin detaches from actin with an effective detachment rate, *k*_*det*_.

A single myosin head (*N* = 1) moves an actin filament a distance, *d*, with a working step every time it completes one actin–myosin ATPase reaction cycle (64, 65, 66) (Fig. 1, *A* and *B*). In theory, the speed at which one myosin head can move an actin filament is *V* = *d*∙*v*, where *v* is the actin–myosin ATPase rate (2, 4, 35). *V* doubles when two myosin heads are moving the same actin filament, triples for three myosin heads, and for N myosin heads is in theory

(2) $$V_{att}\left(N\right)=v\left(N\right)\cdot d$$

At saturating [ATP], *v*(*N*) is limited by *k*_*att*_ (24), and so we refer to *V*_*att*_ as being attachment limited (31) (Fig. 1*C*). At low *N*, *v*(*N*) increases linearly as *N*·*k*_*att*_, and at sufficiently high *N*, *v*(*N*) saturates at *N*_*actin*_·*k*_*ws*_, where *N*_*actin*_ is the number of myosin-binding sites per 1 μm actin filament. We use 1 μm because it is roughly the length above which segmental actin movements become redundant, a length beyond which the force generated by a given myosin head is not transmitted (32, 35). Thus, with saturation of myosin-binding sites on actin, *V*_*att*_(*N*) saturates at a maximum velocity, *V*_*max*_, of *k*_*ws*_·*N*_*actin*_·*d*.

This simple kinetic model for saturation of *V*(*N*) does not take into consideration the mechanical effects of actin-bound myosin heads that impose mechanical loads against actin sliding at high *N*. The probability, *P*(*N*), that actin-bound myosin heads stall actin sliding increases with increasing *N*, and when *P*(*N*) = 1, actin movement can only occur with the detachment of resistive myosin heads at which point *V* reaches a detachment limit (33, 67). In general, the detachment limit occurs when actin-bound myosin heads are stretched a distance *L* before actin movement is stalled at which point actin movement can only resume with the detachment of resistive head(s) at a rate *k*_*det*_ (67). The time it takes an actin filament to move to the point of stall, *L*, is *L*/*V*_*att*_, and once stalled the average time it takes resistive head(s) to detach from actin is 1/*k*_*det*_. Thus the detachment-limited *V* is $L/\left(\frac{1}{k_{det}}+\frac{L}{k_{cat}\cdot N\cdot d}\right)$ (Fig. 1*B*) (33), which for large *N* becomes

(3) $$V_{det}=L\cdot k_{det}$$

One goal of this study is to determine the *N*-dependence of the relative contributions of attachment (Equation 2) and detachment kinetics (Equation 3) to actin sliding velocities. Because definitions of *P*(*N*) and *L* are model dependent, achieving this goal requires discriminating between two fundamentally different models: the independent force generator model and the collective force model (see Discussion for a more detailed description of model differences).

According to the independent force generator model, actin movement stalls when one myosin head is strongly bound to actin and displaces it a distance *d*. *P*(*N*) is then the probability that at least one myosin head is strongly bound to actin, and *L* = *d* (30, 67, 68) (Fig. 1*B*). Equation 3 becomes $V_{det}=\;k_{det}\cdot d$ (Fig. 2*A*) and the *N*-dependence of *V* is *V*(*N*) = *V*_*det*_·*P*(*N*) (32).

According to a collective force model (Fig. 2*C*) actin movement stalls when the compliant linkages associated with myosin heads strongly bound to actin are collectively displaced a distance *L* by the working steps of *N* myosin heads before reaching a stall force, *F*(*N*), which increases linearly with *N* (16). Thus, *P*(*N*) is the probability that a collectively generated internal stall force is reached, and *L* = *F*(*N*)/*κ*, where *κ* is the effective stiffness of the compliant linkages associated with strongly bound heads. According to this model, *V*(*N*) = *V*_*det*_·*P*(*N*) + *V*_*att*_·(1 − *P*(*N*)) (Fig. 2*C*). Based on our recent observation that the myosin S2 tether significantly increases *L* in a myosin filament motility assay, we previously developed an idealized model in which the compliant linkage was infinitely compliant with a hard stop when stretched a distance *L* (the length of the tether) from which we developed an analytical expression (Fig. 2*B*) for *P*(*N*) (33). We refer to this as a collectively displacement model. In general, however, a more realistic model requires strain-dependent kinetics and collective force generation in stretching compliant linkages. For this we develop a simple discrete state computational model for collective force generation (below).

### Collective force computational model

Our collective force model is based on the ATPase kinetic scheme in Figure 1*A* with forward and reverse rate constants set to values consistent with those measured in skeletal muscle myosin. A single elastic element of stiffness κ is collectively displaced by myosin heads a distance 8 nm with each actin–myosin weak-to-strong binding step and 2 nm with each ADP release step. Strain-dependent kinetics are incorporated by multiplying the rate constants for each of the two mechanochemical steps by exp(−*w*/k_B_T), where *w* is the work performed displacing the elastic element. Here, k_B_ is Boltzmann’s constant and T is temperature. Monte Carlo simulations were run with 1-μs time steps. Simulations were run either without saturation kinetics (assuming an infinite number, *N*_*actin*_, of actin-binding sites per micrometer of actin) or with saturation kinetics (assuming an infinitely high weak-binding affinity, *K*, and a fixed number, *N*_*actin*_, of actin-binding sites per micrometer of actin).

## Data availability

All data are contained within the article.

## Conflict of interest

The authors declare that they have no conflicts of interest with the contents of this article.
